# Identification and Characterization of the Caspase-Mediated Apoptotic Activity of *Teucrium mascatense* and an Isolated Compound in Human Cancer Cells

**DOI:** 10.3390/molecules24050977

**Published:** 2019-03-11

**Authors:** Neena Gopinathan Panicker, Sameera Omar Mohammed Saeed Balhamar, Shaima Akhlaq, Mohammed Mansour Qureshi, Tania Shamim Rizvi, Ahmed Al-Harrasi, Javid Hussain, Farah Mustafa

**Affiliations:** 1 Department of Biochemistry, College of Medicine & Health Sciences, United Arab Emirates (UAE) University, Al Ain 20000, UAE; ngpanicker@uaeu.ac.ae (N.G.P.); mlt1987@hotmail.com (S.O.M.S.B.); 201570084@uaeu.ac.ae (S.A.); mohammad.qureshi@uaeu.ac.ae (M.M.Q.); 2 Natural and Medical Sciences Research Center, University of Nizwa, Nizwa, Oman; taniarizvi722003@gmail.com (T.S.R.); aharrasi@unizwa.edu.om (A.A.-H.); 3 Department of Biological Sciences & Chemistry, College of Arts and Sciences, University of Nizwa, Nizwa, Oman; javidhej@unizwa.edu.om

**Keywords:** *Teucrium mascatense*, natural plant products, anticancer activity, breast and cervical cancer, apoptosis, caspases

## Abstract

Plants of the genus *Teucrium* (Lamiaceae or Labiatae family) are known historically for their medicinal value. Here, we identify and characterize the anticancer potential of *T. mascatense* and its active compound, IM60, in human cancer cells. The anti-proliferative effect of a *T. mascatense* methanol extract and its various fractions were analyzed in MCF-7 and HeLa cells in a dose- and time dependent manner. The dichloromethane fraction (TMDF) was observed to be the most effective with cytotoxicity against a more expanded series of cell lines, including MDA-MB-231. A time and dose-dependent toxicity profile was also observed for IM60; it could induce rapid cell death (within 3 h) in MCF-7 cells. Activation of caspases and PARP, hallmarks of apoptotic cell death pathways, following treatment with TMDF was demonstrated using western blot analysis. Inversion of the phosphatidylserine phospholipid from the inner to the outer membrane was confirmed by annexin V staining that was inhibited by the classical apoptosis inhibitor, Z-VAK-FMK. Changes in cell rounding, shrinkage, and detachment from other cells following treatment with TMDF and IM60 also supported these findings. Finally, the potential of TMDF and IM60 to induce enzymatic activity of caspases was also demonstrated in MCF-7 cells. This study, thus, not only characterizes the anticancer potential of *T. mascatense*, but also identifies a lead terpenoid, IM60, with the potential to activate anticancer cell death pathways in human cancer cells.

## 1. Introduction

Cancer is the second major cause of mortality worldwide, responsible for an estimated 9.6 million deaths in 2018, and accounting for one out of every six deaths [1]. This burden is expected to rise from 18.1 million worldwide to 29.5 million by 2040 [2]. The economic burden of cancer is no less, where only in the US, it was estimated to be nearly $1.16 trillion in 2010 alone [3]. The use of plant-derived compounds for cancer therapy is widely prevalent, with more than 60% of clinically approved anticancer drugs being derivatives of medicinal plants [4,5]. However, surprisingly, only 6% of the nearly 250,000 plant species known to exist have been screened for their biological activity [6]. Based on estimates of the World Health Organization (WHO), around 80% of the world’s population depends on herbal medicines, especially natives of Latin America, Asia, and Africa [7], and more than 3000 plant species are currently known to treat cancer [8]. Considering the wealth of untapped knowledge available in the realm of traditional/alternative medicine, there is an urgent need to use advanced screening methods to identify and characterize the anticancer potential of plant extracts [9]. Interestingly, most of the therapeutic drugs currently used in the treatment of breast cancer, the most frequent cause of cancer and cancer-related mortality among women [2], are originally derived from plants [7]. These include paclitaxel and docetaxel (*Taxus baccata*), etoposide (*Podophyllum peltatum*), camptothecin (*Camptotheca acuminata*), and vinblastine and vinorelbine (*Vinca rosea*). In fact, results from recent clinical trials have established docetaxel as the most active single agent in the treatment of advanced metastatic breast cancer, either as first or second-line therapy [10]. Therefore, the skepticism often associated with the translation of compounds derived from plant extracts into commercially viable drugs is unsubstantiated.

*Teucrium mascatense* (*T. mascatense*) is a medicinal plant of the genus *Teucrium*, belonging to Lamiaceae or Labiatae family. The *Teucrium* genus is comprised of around 300 species distributed over central Europe, Western Asia, the Mediterranean region, North Africa, and the Arabian Peninsula [11,12,13,14,15,16,17]. The medicinal value of *Teucrium* species has been known since the times of Socrates and Jalinous, and plants belonging to this genus have been used in both traditional and modern medicine owing to their bioactive constituents [15,16,17,18,19]. Species of *Teucrium* are known to contain tannins, glycosides, phenols, steroids, and terpenoids, with strong biological activities, such as antibacterial, antipyretic, anti-inflammatory, anti-diabetic, anti-spasmodic, analgesic, lipolysis, and antioxidant actions [19,20,21,22,23,24,25,26,27,28,29,30,31]. One species of *Teucrium* from the same family, found in areas like Sardinia and Baronia of Siniscola, has been used as an antimalarial agent [32].

Not much is known about the anticancer potential of *T. mascatense.* Most of the anticancer studies on this plant have been done on *Teucrium polium* (*T. polium*), one of the most widely studied plants of the *Teucrium* genus [33]. It has been shown to be an effective and safe chemo-sensitizing agent as it can potentiate the anti-proliferative and apoptotic effects of various chemotherapeutic drugs, including vincristine, vinblastine, and doxorubicin [34]. *T. polium* may also have anticancer potential that can be attributed to the presence of flavonoids and diterpenoids [19,35]. In addition, secondary metabolites present in this species have been shown to have toxic effects against cancer cells [36,37,38]. Kandouz et al., have shown that extracts of *T. polium* can not only inhibit proliferation of prostate cancer cells, but also inhibit their invasion and motility by altering the expression and localization of E-cadherin and catenins [39]. Finally, a recent study using concentrates of *T. polium* in rats has shown significant anticancer activity against hepatocellular carcinomas [40].

Compared to these, there is a dearth of literature on the anticancer potential of *T. mascatense.* Initial screening of the *T. mascatense* plant extracts showed that it could inhibit growth of human breast cancer cells [41]. Thus, the aim of the current study was to characterize this anticancer potential of *T. mascatense* further in a comprehensive manner. Our work demonstrates that *T. mascatense* can induce apoptotic activity in human breast cells. Furthermore, we go on to demonstrate that an active compound isolated from *T. mascatense* has anti-proliferative and anti-apoptotic activity against breast cancer cells in vitro, leading to the identification of a potential lead compound in the search for natural compounds against cancer.

## 2. Results

### 2.1. Crude Methanolic Extract of T. mascatense and Some of Its Fractions Induce Cytotoxic Effects in Human Normal and Cancer Cell Lines

To determine the anticancer potential of *T. mascatense*, its methanol extract (TMME) along with five of its organic/aqueous fractions were analyzed for their effects on cancer cell proliferation, as described in the Materials and Methods section. The different organic/aqueous fractions of the crude methanol extract were tested to determine which solvents were better at isolating the relevant biologically active content of the plant [42]. The initial screening was limited to two types of human cancer cell lines: MCF-7, a breast cancer cell line [43], and HeLa, a cervical carcinoma cell line [44] (Table 1). This allowed us to determine the general potential of these extract/fractions to affect cell proliferation of two different types of human cancer cell lines. The samples were analyzed at four different concentrations (25, 50, 125, 250 µg/mL), at 24 and 72 h post-treatment. After 24 h of treatment, TMME and two of its organic fractions prepared by using dichloromethane and *n*-hexane solvents (TMDF and TMHF), were observed to be effective against MCF-7 cell line by inducing ≥20% inhibition of cell proliferation at a concentration of 250 µg/mL. None of these three showed cytotoxic effects on HeLa cells 24 h post treatment. After 72 h, TMDF, TMME, and TMHF showed ≥20% cytotoxic effects on the MCF-7 cell line at concentrations of 125 and 250 µg/mL, and were also active against the HeLa cell line at a dose of 250 µg/mL (Table 1).

Based on our experience with the anti-cancer potential of crude methanol extracts and their different fractions from several other plants where the dichloromethane solvent was the most consistent in its ability to induce cytotoxic effects in both MCF 7 and HeLa cell lines, TMDF was chosen for further testing in a more expanded series of cell lines, including MCF-10A and MDA-MB-231. MCF10A is a normal human mammary epithelial cell line [45] and was used to allow comparison of the effect of TMDF on normal versus cancer cell lines, while MDA-MB-231 is a cell line from a triple receptor negative breast cancer tissue [46]. Such breast cancers are much harder to treat due to their inability to respond to therapies directed against hormone receptors [47]; thus, this cell line allowed us to test for natural compounds that may have anti-proliferation activity against them.

**Figure 1:**
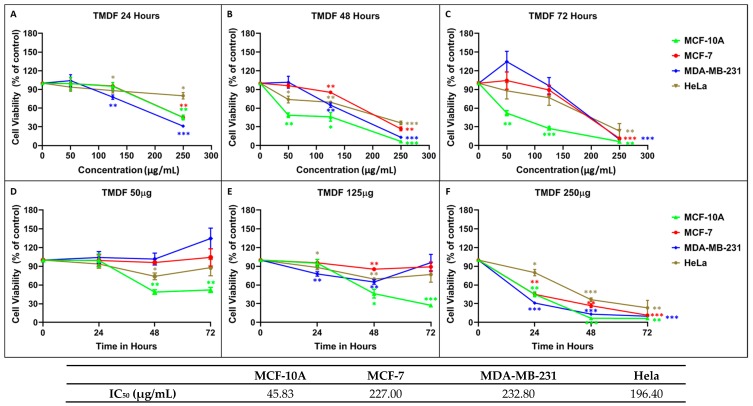
Cytotoxic effects of different concentration of *T. mascatense* dichloromethane fraction (TMDF) using MTT assay on MCF-10A, MCF-7, MDA-MB-231, and HeLa cells after (**A**,**D**) 24, (**B**,**E**) 48, and (**C**,**F**) 72 h post treatment. * indicates statistically significant differences between the DMSO- and TMDF-treated samples (* *p* < 0.05; ** *p* < 0.01 but > 0.001; *** *p* < 0.001). The IC_50_ values (in µg/mL) for the 72-h time points are shown in the table below the figure.

The MTT assay was performed on all the cell lines chosen in a dose-dependent manner using three different concentrations of extract/fractions (50, 125, 250 µg/mL) after 24, 48, and 72 h of treatment. Figure 1 shows the dose-dependency of each cell line and the time course of cell death observed after normalizing cell proliferation to the effects of DMSO alone (the solvent used for solubilizing TMDF). As can be seen, TMDF could induce cytotoxic effects in both the breast and cervical cancer cell lines in a statistically significant manner (Figure 1). However, the normal breast epithelial cell line, MCF-10A, was the most sensitive to TMDF, while the three cancer cell lines showed comparable dose response to TMDF (Figure 1C). Calculation of the dose that caused 50% inhibition of proliferation (IC_50_) confirmed these observations. As can be seen from the table in Figure 1, the IC_50_ value for the 72-h time point for MCF-10A was the least (45.83 µg/mL), followed by that for HeLa (196.4 µg/mL), MCF-7 (227 µg/mL) and MDA-MB-231 cells (232.8 µg/mL). On the other hand, time course analysis of cell viability revealed that the cytotoxicity profile for the normal MCF-10A cells was comparable to the other two breast cancer cell lines, MCF-7 and MDA-MB-231, while the HeLa cells were the slowest in response to TMDF-induced cell death (Figure 1F).

To eliminate the possibility of the observed cytotoxicity being due to the organic solvents used in the extraction process, MTT assay was also conducted on the three cancer cell lines (MCF-7, MDA-MB-231, and HeLa) using only the individual solvents starting at 1:500 dilution. None of the organic solvents showed any cytotoxicity on the tested cell lines (data not shown). This was further validated by nuclear magnetic resonance (NMR) analysis of the extract and fractions which detected no residual organic solvents in these samples (data not shown). These observations, along with the fact that only three of the five organic extract/fractions showed cytotoxicity on the tested cell lines, (Table 1) confirms that the detected anti-proliferative effects on the cell lines were not due to any lingering solvents that may have been left in the extract/fractions; rather, it was due to the compounds present in the extract and fractions themselves.

### 2.2. TMDF Activates Key Apoptotic Proteins in Breast Cancer Cells

**Figure 2:**
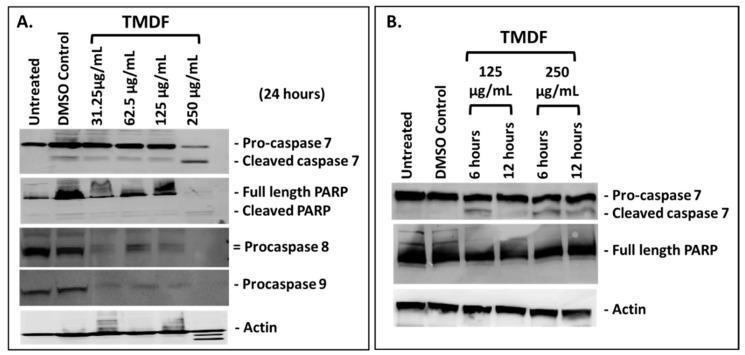
Western blot analysis of MCF-7 cells treated with the dichloromethane fraction of *T. mascatense* (TMDF): (**A**) for 24 h, (**B**) for 6 and 12 h at the indicated concentrations followed by detection of the specified caspases or poly ADB ribose polymerase (PARP) proteins. Actin antibody was used as a loading control. The “DMSO control” sample contains the same amount of DMSO as present in the 250 µg/mL sample of TMDF.

Next, we determined whether the cytotoxicity being observed in the cancer cell lines was due to apoptosis, the primary cell death pathway activated by anticancer compounds [7,48]. The activation of various caspases and downstream effector protein PARP (poly ADB ribose polymerase) are hallmarks of apoptosis [49]. Thus, the classical technique of western blot analysis was used to detect activation of these proteins involved in apoptosis 24 h post treatment with different concentrations of TMDF. As shown in Figure 2A, the expression and cleavage of caspase 7 and PARP proteins was successfully detected, demonstrating that TMDF had the potential to activate a caspase-dependent mechanism of inducing apoptosis in MCF-7 cells. Some degradation of actin could be observed at the 250 µg/mL concentration, suggesting late stages of apoptosis when actin degradation can be expected [50]. Activation of caspase 8 and 9 could also be observed by the steady disappearance of the procaspase 8 and 9 bands; however, the actual cleaved products of these caspases could not be detected (Figure 2A). This is most likely due to the well-known labile nature of the activated caspase cleavage products that requires correct timing and concentration to be visible and the poorer specificity of the antibodies for the cleaved products [51].

To ensure that the caspase cleavage being observed was reproducible and not a reflection of generalized cytotoxicity from TMDF, the westerns for caspase 7 and PARP were repeated at earlier time points (6 and 12 h) on cell lysates generated from MCF-7 cells treated with 125 and 250 μg/mL of TMDF. As can be seen in Figure 2B, caspase 7 cleavage could be detected under these conditions where actin was not degraded, confirming that the caspase 7 cleavage observed earlier was bona fide and not due to a generalized cytotoxicity induced by TMDF. Interestingly, under these conditions, PARP cleavage was not visible, most likely due to the early time points studied since PARP cleavage is a later event in apoptosis [49].

### 2.3. TMDF Can Induce Apoptosis in MCF-7 Cells

**Figure 3:**
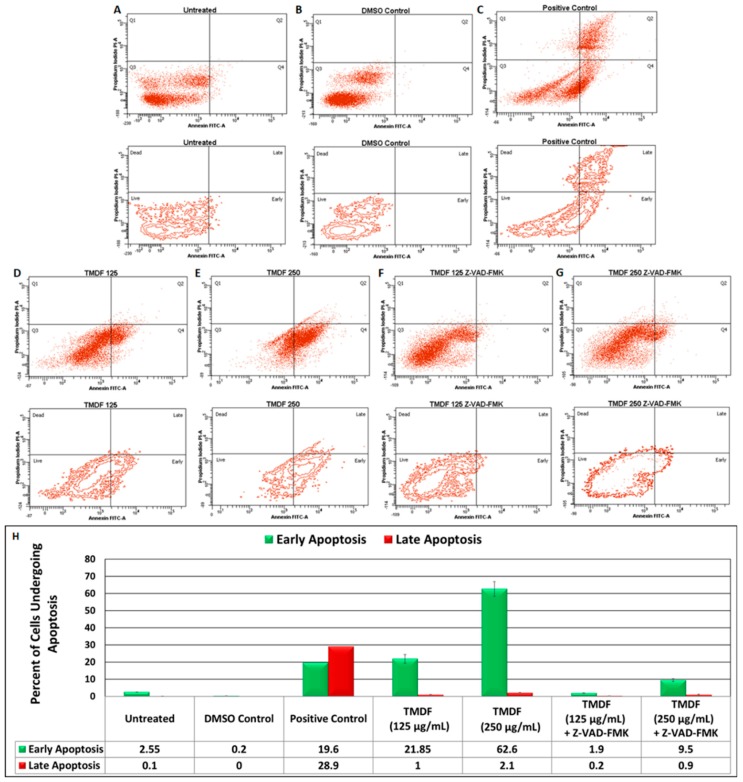
Flow cytometric analysis of Annexin V and propidium iodide-stained MCF-7 cells treated with dichloromethane fraction of *T. mascatense* (TMDF). MCF-7 cells were treated with 125 and 250 µg/mL of TMDF for six hours. (**A**) Untreated cells; (**B**) DMSO control; (**C**) Cells treated with *Boswellia sacra*, a known inducer of apoptosis (positive control) [52]; (**D**) Cells treated with 125 µg/mL of TMDF; (**E**) Cells treated with 250 µg/mL of TMDF; (**F**) Cells pretreated with 20 µM of Z-VAD-FMK followed by six hour treatment with 125 µg/mL of TMDF; (**G**) Cells pre-treated with 20 µM of Z-VAD-FMK followed by six hour treatment with 250 µg/mL of TMDF. (**H**) Column graph that plots the percentage of cells that shift into the early (green bars) and late (red bars) of apoptosis upon the various treatments mentioned above. The “DMSO control” sample contains the same amount of DMSO as present in the 250 µg/mL sample of TMDF.

Another hallmark of apoptosis is inversion of the inner leaflet of the plasma membrane, exposing the proteins and phospholipids that reside on the inside of the plasma membrane phospholipid bilayer without compromising membrane integrity [49]. This is considered an early event observed in cells undergoing apoptosis in which the cell viability is maintained despite the inversion of the plasma membrane [49]. Therefore, we tested for the ability of TMDF to induce this classical effect on treated cells using the well-established Annexin V/propidium iodide (PI) staining assay and flow cytometry [49]. As can be seen from Figure 3, compared to the untreated cells, DMSO treatment did not affect the viability of the cells (Figure 3A,B). This is in contrast to our positive control (*Boswellia sacra* essential oil) that has a potent ability to induce apoptosis in a rapid manner (Figure 3C) [52]. Treatment of MCF-7 cells with *Boswellia sacra* essential oil led to shift of the cells into the Annexin V-positive, but PI negative quadrants (19.6%), revealing the induction of early apoptotic events (Figure 3C) as well as late apoptosis (28.9%) where the Annexin V population was PI positive (Figure 3H). In comparison, treatment of MCF-7 cells for six hours with 125 µg/mL of TMDF led to 21.85% of the cells to move into the early apoptosis phase (Figure 3D), while treatment with 250 µg/mL lead to a further increase in apoptosis induction with 62.6% of the cells moving into early apoptosis phase and 2.1% into late apoptosis (Figure 3E,H). This dose-dependent increase in cells entering the early apoptotic stage reveals that TMDF has the capability to induce apoptosis in MCF-7 cells.

To confirm whether this was indeed apoptosis, we further tested the ability of the classical apoptosis inhibitor, Z-VAK-FMK to inhibit this process. As can be seen, pretreatment of the MCF-7 cells with 20 µM Z-VAK-FMK led to a significant inhibition of apoptosis in both 125 and 250 µg/mL TMDF-treated cells (Figure 3F,G) with only 1.9 and 9.5% of the cells shifting into the early apoptotic phase compared to 21.85 and 62.6% of the cells without the inhibitor (Figure 3H). These results conclusively show that TMDF has the ability to induce apoptosis in breast cancer cells.

### 2.4. Effects of TMDF on the Morphological Properties of Treated Cells

**Figure 4:**
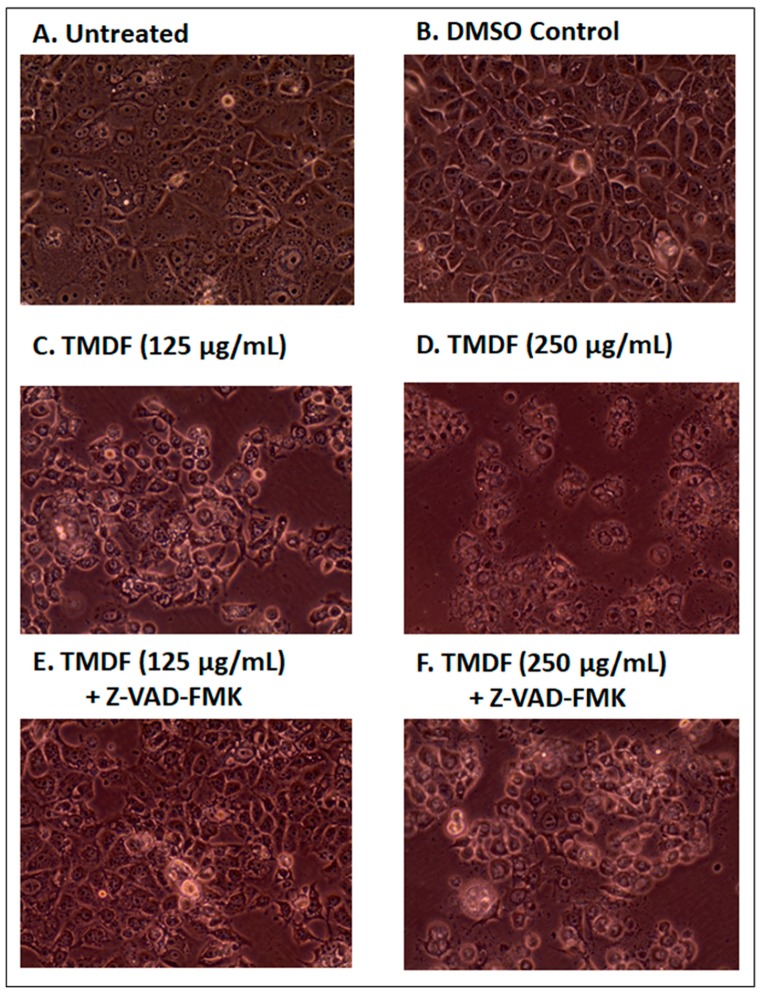
Photomicrographs of MCF-7 cells 24 h after treatment with: (**A**) media alone; (**B**) DMSO; (**C**) 125 µg/mL, and (**D**) 250 µg/mL *T. mascatense* dichloromethane fraction (TMDF). Panels **E** and **F** show the results after a 2-h pretreatement of the cells with 20 µM of the apoptosis inhibitor Z-VAD-FMK before being treated with either (**E**) 125 µg/mL or (**F**) 250 µg/mL of TMDF for 24 h. Magnification: 400×. The “DMSO control” sample contains the same amount of DMSO as present in the 250 µg/mL sample of TMDF.

Since the western blot and Annexin V/PI analyses of cells treated with TMDF suggested induction of apoptosis, we next analyzed the effect of TMDF treatment on the morphological characteristics of MCF-7 cells. Such changes started to appear within one day of extract treatment. Figure 4 shows the effect of TMDF on MCF-7 cells 24 h post treatment with 125 and 250 µg/mL of TMDF (Figure 4C,D). As can be seen, differences were observed in cell morphology of the treated MCF-7 cells, including appearance of cell rounding, cell shrinkage, and detachment of cells from other cells and the plate. Importantly, these changes were absent in untreated (Figure 4A) or DMSO-treated cells (Figure 4B).

Next, MCF-7 cells were pretreated with Z-VAD-FMK prior to exposure to TMDF to determine whether the morphological changes associated with apoptosis could be reduced or eliminated upon the inhibitor treatment. As can be seen in panels E and F of Figure 4, pretreatment of the TMDF-treated cultures with Z-VAD-FMK essentially eliminated (Figure 4E) or reduced (Figure 4F) the appearance of the apoptosis-specific morphological changes in cultures treated with 125 and 250 µg/mL of TMDF. These data support our conclusion that TMDF can activate apoptosis and further suggest that apoptosis is probably the major mechanism of cell death in cultures treated by TMDF.

### 2.5. TMDF Activates Caspase Activity in Human Breast Cancer Cells

**Figure 5:**
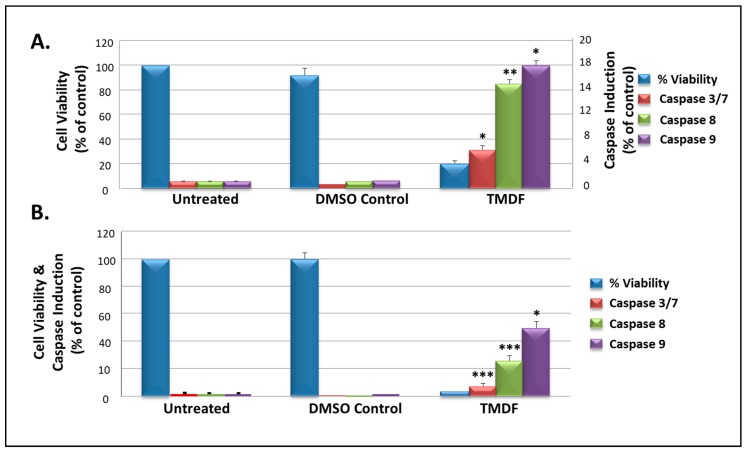
Viability and caspase activity in MCF-7 cells treated with 250 µg/mL of *T. mascatense* dichloromethane fraction (TMDF) for 48 h, as shown in two independent experiments (panels **A** and **B**). * indicates statistically significant differences between the control and treated samples (* *p* < 0.05; ** *p* < 0.01 but > 0.001; *** *p* < 0.001). The “DMSO control” sample contains the same amount of DMSO as present in the 250 µg/mL sample of TMDF.

To confirm the potential of TMDF to activate caspases, the Promega caspase GLO assay was used to detect the presence of their enzymatic activity. Towards this end, MCF-7 cells were treated with 250 µg/mL TMDF and tested for the induction of caspase 3/7, 8, and 9 enzymes. Figure 5 shows two independent experiments testing the ability of TMDF to inhibit cell proliferation in MCF-7 cells (blue bars) in parallel with its ability to induce caspase activity. In both experiments, a low cell viability (blue bars) was associated with the induction of high levels of caspase 8 (green bars) and 9 (purple bars) as well as caspase 3/7 (maroon bars) in a statistically significant manner.

Since MCF-7 cells do not express any caspase 3 [53], any induction of caspase 3/7 in this system is most likely due to the induction of caspase 7. Results obtained for the induction of caspases were normalized against the number of viable cells in culture (obtained using the Cell Titer-GLO luminescent cell viability assay) to ensure that the enzymatic activity being measured was taking into consideration the ensuing cell death being observed (Figure 5). These observations correlated well with the MTT results presented earlier (Figure 1). Overall, these results strengthen our conclusion that TMDF contains bioactive molecules that can cause cell death via caspase-dependent apoptosis.

### 2.6. Test of the Anti-Proliferation Effect of IM60 on Breast Cancer Cells

Next, we wanted to study whether a lead compound could be identified from *T. mascatense* with potential anticancer activity. Medicinal plants and their metabolites have an important role in cancer treatment [47,48,54] and their natural or synthetic derivatives could play crucial roles in preventing, slowing, or reversing cancer development [55,56]. Therefore, we screened one purified compound isolated from the dichloromethane fraction of the crude methanolic extract of *T. mascatense*, IM60, to determine its potential anticancer activity [57].

**Figure 6:**
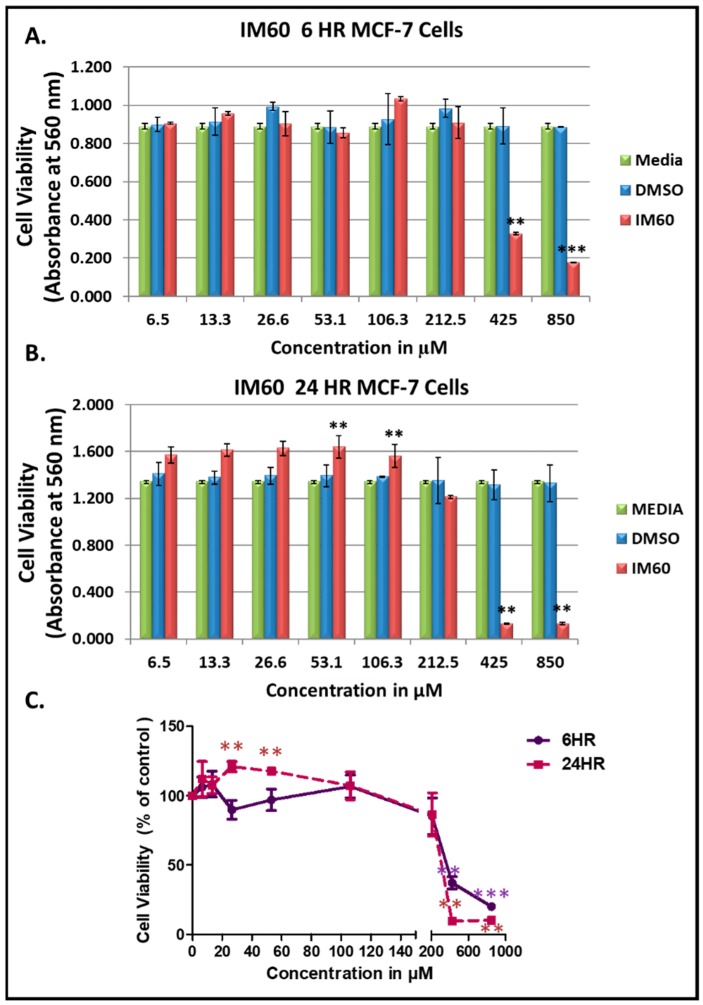
Test of IM60 using MTT assay on MCF-7 cells: (**A**) 6 h post treatment, and (**B**) 24 h post treatment. (**C**) Dose-dependent effect of IM60 on cell viability as a percentage of DMSO-treated cells. * indicates statistically significant differences between the DMSO control and IM60 treated samples (** *p* < 0.01 but > 0.001; *** *p* < 0.001). IM60 was tested twice in the MTT assay in triplicates.

**Figure 7:**
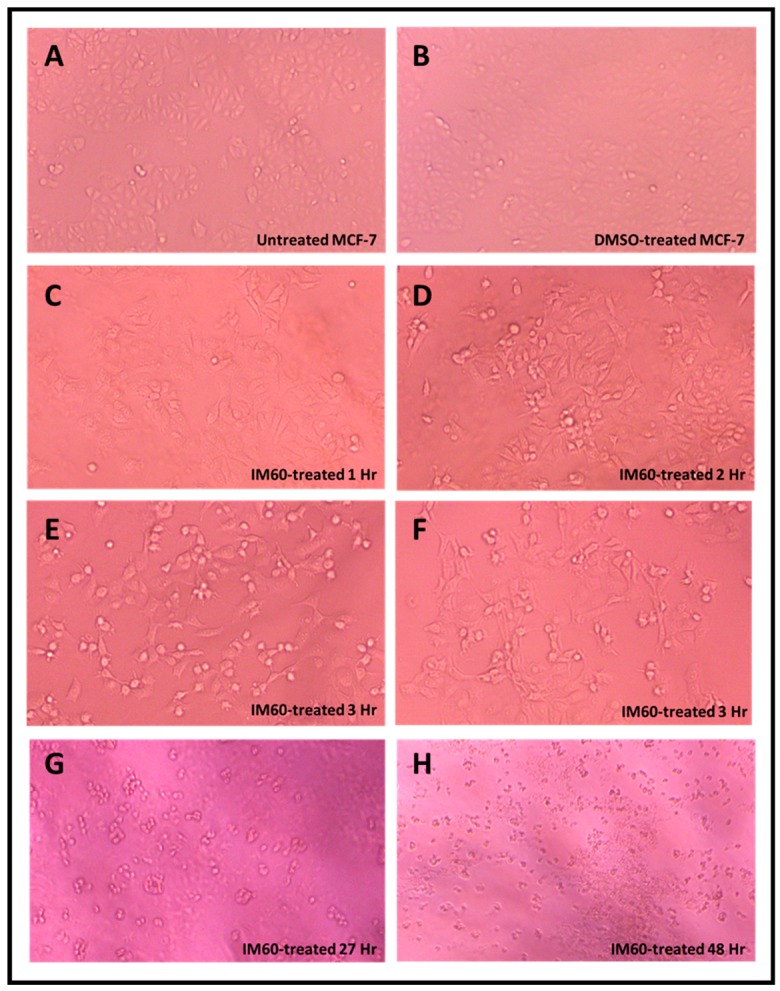
Photomicrographs of MCF-7 cells treated with IM60 compound at 0.425 mM along with its DMSO control for various time points post treatment (magnification 400×). Panels **A**–**F** are from one experiment, while Panels **G** and **H** are from another experiment that was conducted for a longer time period.

Figure 6 show the results of the dose-dependent effect of IM60 on MCF-7 cells using the MTT assay. The compound was observed to be effective with more than 90% of the MCF-7 cells being killed after 24 h of treatment at a concentration starting at 425 µM (Figure 6). Some activation of cell proliferation was observed at lower concentrations in the 24-h test; however, it was not observed in the 6-h test, suggesting that it could be an artefact. The IC_50_ value was calculated to be 403 µM for the 24-h time point. The earliest differences in cell morphology could be observed at three hours post treatment, eventually leading to complete cell death (Figure 7). Cell shrinkage and rounding and detachment from other cells was noted early on with a loss of cell numbers, which again suggested apoptosis as a possible mechanism of cell death induced by IM60 (Figure 7).

### 2.7. IM60 Can Induce Caspase Activity in MCF-7 Cells

**Figure 8:**
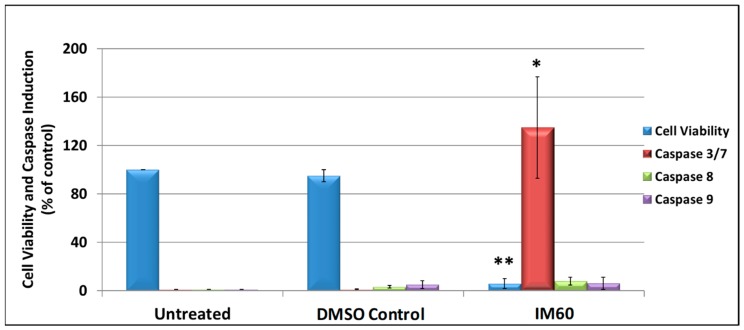
MCF-7 cells were treated with 425 µM of IM60 for 6 h and then assayed for cell viability and induction of different caspase enzyme activities (3/7, 8, and 9). * indicates statistically significant differences between the DMSO-treated and IM60-treated samples (* *p* < 0.05 but > 0.01; ** *p* < 0.01). The “DMSO control” contains the same amount of DMSO as present in the IM60-treated sample.

To determine whether IM60 had the potential to induce functional caspase activity, it was tested in the caspase GLO assay conducted on MCF-7 cells at the effective concentration of 425 µM for 6 h. As expected, in comparison to DMSO-treated cells, cell viability was reduced by more than 90% in IM60-treated cells (*p* < 0.002), confirming its cytotoxic effect on MCF-7 (Figure 8). In addition, a statistically significant activation of the effector caspase 7 was noted (*p* < 0.03); however, no significant activation was observed of the initiator caspases 8 or 9. These data suggest that IM60 can cause inhibition of MCF-7 cell proliferation and may have the potential to activate caspase activity as well (Figure 8). Despite the high concentration needed for observing these effects, these are encouraging results since test of a compound and its several derivatives from another medicinal plant tested in parallel did not result in cytotoxicity, and in fact resulted in activation of cancer cell proliferation (personal observations).

## 3. Discussion

This study assessed the anticancer potential of *T. mascatense* by analyzing its different organic and aqueous extract/fractions, including *n*-hexane, dichloromethane, ethyl acetate, *n*-butanol, methanol, and water. Our results revealed that only three of the organic extract/fractions tested (*n*-hexane, methanol, and dichloromethane) had anti-proliferation activity against several human cell lines, including MCF-7, HeLa, and/or MDA-MB-231 (Table 1 and Figure 1), confirming the earlier preliminary test of these extract/fractions [41]. However, we did not find any inhibitory effect of the aqueous extract on either MCF-7 or HeLa cell proliferation, while this fraction showed moderate cytotoxic activity against MDA-MB-231 in the previous study [41]. This difference could be due to the cell line tested. The dichloromethane fraction (TMDF), on the other hand, showed cytotoxic potential against all three cancer cell lines tested, two breast cancer and one cervical cancer (Table 1 and Figure 1). The mechanism of cell death used by TMDF was demonstrated to be apoptosis which was caspase-dependent (Figure 2, Figure 3, Figure 4 and Figure 5). Furthermore, test of the cytotoxicity of an active constituent of TMDF, IM60, revealed a time and dose-dependent effect (Figure 6); it could induce rapid cell death (within 3 h) with morphological changes reminiscent of apoptosis (Figure 7), and activate caspase 7 enzymatic activity (Figure 8).

Thus, this study adds *T. mascatense* as another *Teucrium* species with anticancer potential. In particular, one study has compared the anti-proliferative effects of six common *Teucrium* species on cell viability of colon cancer cells HTC-116, as well as *T. polium* [58]. Methanolic extracts of all species tested (*T. chamaedrys*, *T. montanum*, *T. arduini*, *T. scordium subsp. Scordium, T. scordium subsp. Scordioides*, *T. polium* and *T. botrys*) inhibited the proliferation of HTC-116 in MTT assays. Interestingly, the IC_50_ values measured at 72 h post treatment fell between 59–253 µg/mL for all except *T. arduini* which is comparable to our results. Using a crude method to detect apoptosis, their data further suggests that all the tested species could induce apoptosis in these cells. Two other studies have recently tested other species of *Teucrium* for anti-proliferative activity for different types of cancer cells. *T. persicum* can potentially induce apoptosis of prostate cancer cells with inhibitory effects on viability of breast and colon cancer cells [59]. It has also been shown to effect cell migration and epithelial cell morphology in the same study. *T. pruinosum*, on the other hand, has been shown to affect the viability of cervical cancer cell line HeLa [60]. These data suggest that the *Teucrium* genus is a rich source of natural anticancer compounds for further investigation.

While the studies mentioned above have tested the effects of extracts from various *Teucrium* species on cancer cell proliferation, their effect on normal cells has not been studied. The cytotoxic effect of TMDF, on the other hand, has been studied on normal cells and was observed to affect the viability of the normal MCF-10A cells also (Figure 1). This is not very surprising since crude extracts are composed of a number of active biomolecules with different potentials to affect cell proliferation in a cell-specific manner. Thus, it is possible that the biomolecules (or their specific combination) responsible for the cytotoxicity for normal and cancer cells differ, potentially belonging to different compounds that can be separated from each other. That is why the pure compound IM60 was tested to determine if it could affect proliferation of cancer cells. Unfortunately, due to its limited availability, we could not test it on normal cells. In future we plan to test this compound more extensively in both normal and cancer cells to characterize its anti-proliferation potential and determine whether its cytotoxicity can be enhanced for cancer cells and modulated differentially in normal and cancer cells via biochemical derivatization.

The various fractions of TMME tested in this study were made in a sequential manner to separate the active constituents of *T. mascatense* selectively, based on polarity [41,57]. It is well known that the choice of the solvent affects the rate of extraction, the type and quantity of phytochemicals extracted, ease of handling of the extracts, and the health hazards associated with the extraction process [42,61,62]. Among the five tested solvents, dichloromethane [63] and *n*-hexane were observed to be the most effective in isolating the active compounds responsible for the anti-proliferation activity observed in the cancer cells. The remaining organic extracts, and especially the aqueous content, were not cytotoxic, indicating that the biochemical entities responsible for anti-proliferation were primarily hydrophobic in nature. Numerous studies have shown chloroform (a trichloromethane) to be effective in extracting anticancer agents from several medicinal plants, such as *Angelica archangelica*, *Nepeta deflersiana*, and *Solanum nigrum* [64,65].

**Figure 9:**
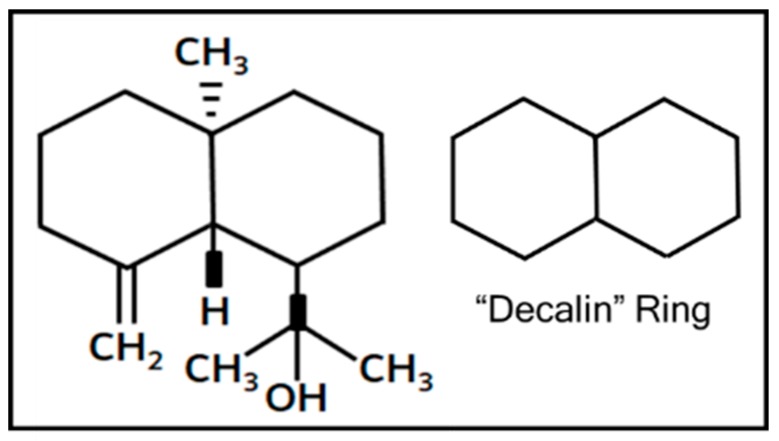
Structure of IM60 with the decalin ring highlighted on the side.

IM60, tested for its anticancer activity in this study, is a sesquiterpene, 1-isopropanol-4a-methyl-8-methylenedecahydronaphthalene, with the structure shown in Figure 9. It was isolated from the dichloromethane fraction of the crude methanolic extract of *T. mascatense* (TMDF) [57]. Sesquiterpenes belong to a large and diverse group of plant-derived bioactive compounds with promising anticancer, anti-inflammatory, antifungal, antibacterial, and immunosuppressive activities [61,66,67]. This is due to the presence of the decalin ring that imparts great structural and functional variety to these compounds [68] (Figure 9). In one study on the anticancer potential of four different species of *Teucrium*, Menichini et al., identified *T. polium* as the best among the four due to the sesquiterpene content of its essential oil which included compounds such as spathulenol, D-cadinene, caryophyllene, etc. [69]. Identification of a novel sesquiterpene with the ability to activate caspase 7 in this study thus adds to the arsenal of new biomolecules being discovered in the fight against cancer.

This study shows that caspase-dependent apoptosis is the mechanism of cell death induced by the dichloromethane fraction of *T. mascatense* and perhaps its active component IM60. Apoptosis as a mechanism of cell death has been observed in other plant-derived anticancer compounds as well [7,48]. Most of them induce cell death that may be intrinsic or extrinsic, and caspase and/or p53-dependent or independent. Levitsky and Dembitsky recently analyzed the effect of a large number of plant extracts on breast cancer cells, and observed apoptosis to be one of the most common mechanisms of inducing cell death [7]. For example, genistein was found to induce apoptosis in MCF 7 and T47D breast cancer cell lines. Similarly, oleuropein aglycone found in extra virgin olive oil increased apoptotic cell death by factors of 1.5, 2.5, and 4 in MCF-5, MCF 7/HER, and SK-Br3 cells, respectively. Even “anticancer diets” and crude preparations from a large variety of vegetables have shown to induce apoptosis in breast cancer cell lines. In a study performed on MCF-7 and MDA-MB-231, the cells were pretreated for 72 h with increasing concentrations of *Brassica olearacea* juice, and results suggested that presence of active compounds from cabbage juices activated both apoptosis and necrotic pathway in the breast cancer cells. Similarly, pro-apoptotic effects of green tea extracts and tea catechins have also been reported in tumor cells, both in vitro and in vivo [7,48].

Our preliminary data suggests that TMDF may have the potential to induce autophagy (data not shown), an important cell survival process that is being implicated as a mechanism that prevents neoplastic transformation of cells as well [70,71]. Due to the crude nature of the extracts/fractions tested in this study, it remains to be determined what other cell death pathways can be induced by TMDF and other extract/fractions of *T. mascatense* to understand the full anticancer potential of this plant species.

In this study, we used a cancer cell line model system to study the anticancer potential of our extracts/fractions. Although breast cancer cell lines, in general, are considered to be crude models of the disease as they may not be able to capture the intra- and inter- tumor heterogeneities [72,73], the fact that MCF-7 and MDA-MB-231 cell lines that were used in our analysis, along with T47D, account for more than two-thirds of cell lines used in preclinical studies analyzing breast cancer drugs [73], renders our analysis to be state-of-the-art.

Large-scale screening of plant extracts for anticancer potential has mostly not been very encouraging. In a study of more than 1000 aqueous and organic extracts from 351 species of Brazilian rain forests, only 11 extracts showed any cytotoxicity in MCF-7 cells at a dose of 0.1 mg/mL [74]. An 8-year study of 7500 South African plant extracts found a total of only 50 active extracts when screened against 60 cell lines; none of them was found to be potent in MCF-7, with only 20 presenting moderate inhibiting activity [75]. One reason why they may have missed detecting such activities could be the timing and dosage of the screening. Despite such discouraging data from large-scale analysis, there is reason to believe that concerted, meticulous efforts in the testing of crude plant extracts in a dose and time-dependent manner can lead to identification of active compounds and reveal cellular mechanisms involved in their anticancer potential, as observed in our study.

## 4. Materials and Methods

### 4.1. Extraction and Isolation of Plant Material

**Figure 10:**
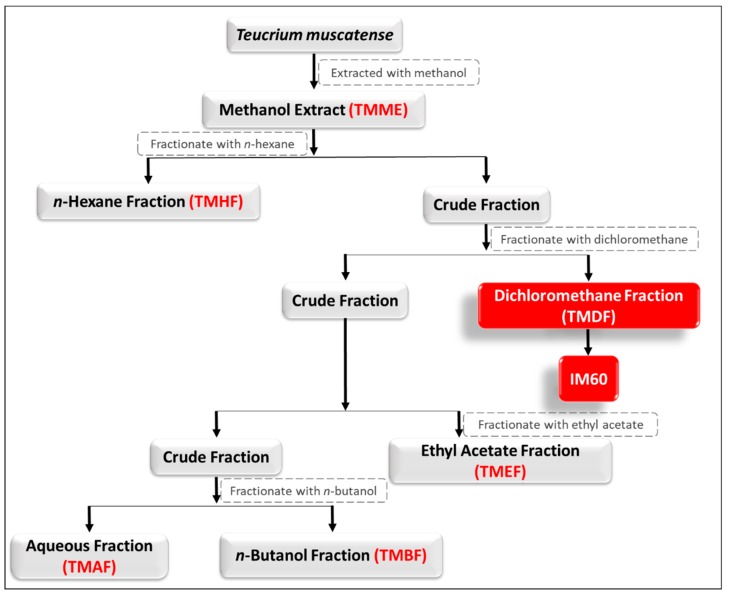
Schematic illustration of the extraction strategy used and isolation of the various fractions and IM60 from the methanolic extract. TMDF and IM60 are highlighted in red as they were the ones characterized in this study in more depth.

*T. mascatense* Boiss (Lamiaceae) was collected in June 2013 from the mountains of *Al-Jabel Al-Akhdar*, Oman. The collection, identification, and extract preparation of the plant has been described previously [41]. A voucher specimen was deposited with the Herbarium of the University of Nizwa, Oman. Briefly, the crude methanolic extract of the whole plant of *T. mascatense* (TMME) was partitioned into five different fractions by solvent-solvent partition with increasing polarity, starting with *n*-hexane (TMHF), dichloromethane (TMDF), a more stable and safer substitute of chloroform with less genotoxic effects [63], ethyl acetate (TMEF), *n*-butane (TMBF), followed by water (TMAF) [41] (Figure 10). IM60 was isolated from the dichloromethane fraction of the crude methanolic extract of *T. mascatense* [57] (Figure 10).

To study the anti-proliferative potential of the various extract/fractions of *T. mascatense*, TMME and four of its fractions, TMHF, TMDF, TMEF, TMBF were solubilized in dimethyl sulfoxide (DMSO), while TMAF was solubilized in water to prepare stock solutions at 50 mg/mL (some at lower concentrations due to solubility issues). The stock solutions were stored at −20 °C till the proliferation assays were performed in different cell lines. A compound from *T. mascatense*, IM60, was isolated [57] and tested for its effect on cell proliferation by dissolving it in DMSO. Using the stock solutions of TMME and its fractions, different dilutions were prepared at twice the final concentration (50, 100, 250, and 500 µg/mL), depending upon the cell line used. A final volume of 100 µL of extract/fractions was added to cells plated in 100 µL media on the day of treatment.

### 4.2. Cell Lines and Culture Medium

Human breast cancer cell lines, MCF-7 (an estrogen and progesterone receptor positive, hormone responsive cell line [43]) and MDA-MB-231 (a triple receptor negative cell line [46]) were cultured in Dulbecco’s Modified Eagles Medium (DMEM)/high glucose medium, supplemented with 10% fetal bovine serum (FBS), 10,000 units/mL penicillin/streptomycin (Pen/Strep), and 50 µg/mL gentamicin, while the human cervical cancer cell line, HeLa [44] was grown in DMEM/high glucose medium supplemented with 7% fetal calf serum (FCS), 10,000 units/mL Pen/Strep, and gentamicin (all reagents by HyClone Laboratories, Inc., Logan, UT, USA). The normal breast cancer epithelial cell line, MCF-10A [45] was cultured in DMEM/F12 medium (DMEM/Hams nutrient mixture) and 5% horse serum supplemented with 10,000 units/mL of Pen/Strep, 10 μg/mL insulin, 20 ng/mL epidermal growth factor (EGF), 0.5 mg/mL hydrocortisone, and 100 ng/mL cholera toxin (all reagents by Sigma-Aldrich, St. Louis, MO, USA). The cell lines were maintained at 37 °C in a 5% CO_2_ humidified incubator.

### 4.3. MTT Cell Proliferation Assay

The cytotoxic effect of the extract/fractions was determined using the [3-(4,5-dimethylthiazol-2-yl)-2-5-diphenyltetrazolium bromide] (MTT) colorimetric method [76]. A dose- and time-dependent analysis of the MTT assay was carried out for all the extract/fractions on specified cell lines. Briefly, cells were cultured in 96-well plates at a density of 5000 cells/well/100 µL media. The cell number was determined empirically after testing the proliferative capacity of MCF-7 and HeLa cells in MTT assays using increasing numbers of cells. Using this data, a cell number range was used which provided a linear signal for either MCF-7 or HeLa cell lines.

For the MTT assay, 24 h after plating, the cells were treated with 100 µL of different concentrations of *T. mascatense* extract/fractions dissolved in DMSO, DMSO alone diluted in media to the same concentration as the extract/fractions (DMSO control), or culture media alone for various time points. To measure proliferation in each well, 25 µL of an MTT stock solution (5 mg/mL) was added, and the plates were incubated for 3–4 h in an incubator (at 37 °C). This was followed by decanting the culture media from the plates, dissolving the formazan crystals formed with 200 µL of DMSO, and measuring the absorbance at 560 nm using a plate reader. Cell viability was measured as the percentage of cells treated with DMSO alone, using same concentrations of DMSO that were used to dissolve the extract/fractions (0.2–1%). The IC_50_ values for all the cell lines were calculated using the non-linear regression method of GraphPad Prism 7.4 software (GraphPad Software, San Diego, CA, USA) for the 72-h DMSO-normalized time points or the 24-h time point for IM60.

### 4.4. Cell Titer-GLO Cell Viability and Caspase-GLO 3/7, 8 & 9 Luminescent Assays

MCF-7 cells were seeded at a density of 5000 cells/well/100 µL in opaque white 96-well plates for tissue culture (Thermo Fisher Scientific, Waltham, MA, USA). After 24 h, cell culture was treated in triplicates with different extract/fractions concentrations or IM60 (in a 50 µL volume) at 37 °C. Cell viability was measured after 48 h using the Cell Titer-GLO Luminescent Cell Viability Assay or the Promega Caspase 3/7, 8 & 9 GLO Assays (Promega Corporation, Fitchburg, WI, USA), according to the manufacturer’s directions. Luminescence was measured using the Infinite M200 Pro Tecan plate reader. Data were plotted as percent cell viability of treated groups compared to DMSO-treated cells, the viability of which was taken as 100%. The experiments with TMDF were performed 2 times with each sample tested in triplicates.

### 4.5. Morphological Studies

To determine morphological changes, cell cultures treated with various concentrations of TMDF or IM60 at different time-points and observed under an inverted light microscope attached to a charged couple device (CCD) camera or the EVOS Cell Imaging System (Thermo Fisher Scientific, Waltham, MA, USA). A comparison was performed between the treated cells and untreated cells or cells treated with similar DMSO concentrations.

### 4.6. Western Blot Analysis

Western blotting of protein lysates was performed to determine the mechanism of cell death of TMDF. The harvested cells treated with various concentrations of TMDF, DMSO, or untreated cells were washed with 1x phosphate-buffered saline (PBS) and lysed using 100 µL of radioimmunoprecipitation assay buffer (RIPA) lysis buffer (10 mM Tris-Cl [pH 8.0], 1 mM ethylenediaminetetraacetic acid (EDTA), 1% Triton X-100, 0.1% sodium deoxycholate, 0.1% sodium dodecyl sulfate (SDS), and 140 mM NaCl) per million cells supplemented with 50 µL of β-mercaptoethanol/mL RIPA and 1 mM of the serine protease inhibitor, phenylmethyl sulfonyl fluoride (PMSF). The lysed cells were spun at 14,000 rpm for 10 min at 4 °C to separate the nuclei, and the supernatants containing the cell extract either used immediately or stored at −80 °C. The quantification of the proteins in the cellular extracts was performed using the Bradford colorimetric assay (Bio-Rad Life Sciences, Hercules, CA, USA) as per manufacturer’s instruction. Total cellular protein lysates (40 µg per lane) were loaded onto 8–12% SDS-polyacrylamide mini gels and blotted onto Protran nitrocellulose membranes (Whatman plc-GE Healthcare, Kent, UK) using standard protocol. The membranes were blocked with 5% low fat dried milk in 1× PBS and 0.1% Tween-20 (PBST) for 1 h and incubated at 4 °C overnight with 1:1000 dilution of various primary antibodies from Cell Signaling Technology (Danvers, MA, USA) against poly (ADP-ribose) polymerase (PARP), caspase 7, caspase 8, and caspase 9, or actin (Sigma-Aldrich, St. Louis, MO, USA) in 1% milk-PBST. The blots were then incubated with the appropriate secondary antibodies (anti-mouse or anti-rabbit) for 1 h and 30 min at room temperature, followed by detection using the Pierce™ ECL Plus Western Blotting Substrate (Thermo Fisher Scientific, Waltham, MA, USA). The chemiluminescent signal was captured using Typhoon FLA 9500 (GE Healthcare, Chicago, IL, USA).

### 4.7. Annexin V/Propidium Iodide Staining

Early events of apoptosis induction were studied using the classical Annexin V/PI staining assay employing flow cytometry. MCF-7 cells were treated with media alone, DMSO, or either 125 or 250 µg/mL of TMDF for six hours in duplicates. To inhibit apoptosis, cells were pretreated with 20 µM of Z-VAD-FMK (Promega Corporation, Fitchburg, WI, USA) before treatment with TMDF. Following treatment, cells were harvested with Accutase (Sigma-Aldrich, St. Louis, MO, USA), a trypsin substitute, and processed for flow cytometry using the Annexin V/PI kit from Becton Dickenson (Franklin Lakes, NJ, USA) as per manufacturer’s instructions.

### 4.8. Statistical Analysis

Statistical analysis was performed using Microsoft Excel (Microsoft, Redmond, WA, USA) or GraphPad Prism version 5 (GraphPad Software, San Diego, CA, USA) or higher software. For each sample, averages were calculated in replicates of three or more, along with their standard deviations (plotted as error bars on the column graphs). Significance of variation between any two groups was assessed using paired, two-tailed student’s *t*-test (* *p* < 0.05; ** *p* < 0.01 but >0.001; *** *p* < 0.001). Since a specific DMSO control was used for each tested extract/fraction, the dose- and time-dependent GraphPad figures were created by normalizing the values obtained for the test groups against their specific DMSO control.

## 5. Conclusions

This study characterized the anticancer potential of *T. mascatense* which resulted in the identification of a potential lead anticancer compound. We found that various organic extract/fractions of *T. mascatense*, especially TMDF, could induce cell death in breast cancer and cervical cell lines. In the breast cancer cells, the cell death was primarily caused by the induction of apoptosis, providing evidence to test its effectiveness and anti-proliferative activity in animal models. Furthermore, a lead sesquiterpene, IM60, was identified from TMDF with the potential to induce cell death in breast cancer cells. It would be valuable to characterize this compound experimentally further (or its derivatives) for its mode of action and conduct in silico docking analysis for breast cancer receptors to determine if it can be specifically targeted against breast cancer cells that are responsive to hormone treatment.

## Figures and Tables

**Table 1:** Summary of the first screening of *T. mascatense* extract/fractions using the MTT assay.

Extract/Fractions	MCF-7 (24/72 HRS) Breast Cancer Cells (µg/mL) *	HeLa (24/72 HRS) Cervical Cancer Cells (µg/mL) *
TMHF	24HR: 250 72HR: 125/250	24HR: X 72HR: 250
TMDF	24HR: 250 72HR: 125/250	24HR: X 72HR: 250
TMEF	X	X
TMBF	X	X
TMME	24HR: 250 72HR: 125/250	24HR: X 72HR: 250
TMAF	X	X

X = Extract/fractions displaying ≤ 20% inhibition of proliferation. * = Numbers denote extract/fractions concentration (in µg per mL) at which ≥ 20% inhibition of proliferation was observed. *Abbreviations*: TMHF: *Teucrium mascatense n*-hexane fraction; TMDF: *Teucrium mascatense* dichloromethane fraction; TMEF: *Teucrium mascatense* ethyl acetate fraction; TMBF: *Teucrium mascatense n*-butanol fraction; TMME: *Teucrium mascatense* methanol extract; TMAF: *Teucrium mascatense* aqueous fraction.
